# Anthelmintic activity of *trans*-cinnamaldehyde and A- and B-type proanthocyanidins derived from cinnamon (*Cinnamomum verum*)

**DOI:** 10.1038/srep14791

**Published:** 2015-09-30

**Authors:** Andrew R. Williams, Aina Ramsay, Tina V. A. Hansen, Honorata M. Ropiak, Helena Mejer, Peter Nejsum, Irene Mueller-Harvey, Stig M. Thamsborg

**Affiliations:** 1 Department of Veterinary Disease Biology, Faculty of Health and Medical Sciences, University of Copenhagen, Frederiksberg, Denmark; 2 Chemistry and Biochemistry Laboratory, School of Agriculture, Policy and Development, University of Reading, Reading RG6 6AT, United Kingdom

## Abstract

Cinnamon (*Cinnamomum verum*) has been shown to have anti-inflammatory and antimicrobial properties, but effects on parasitic worms of the intestine have not been investigated. Here, extracts of cinnamon bark were shown to have potent *in vitro* anthelmintic properties against the swine nematode *Ascaris suum*. Analysis of the extract revealed high concentrations of proanthocyanidins (PAC) and *trans-*cinnamaldehyde (CA). The PAC were subjected to thiolysis and HPLC-MS analysis which demonstrated that they were exclusively procyanidins, had a mean degree of polymerization of 5.2 and 21% of their inter-flavan-3-ol links were A-type linkages. Purification of the PAC revealed that whilst they had activity against *A. suum*, most of the potency of the extract derived from CA. *Trichuris suis* and *Oesophagostomum dentatum* larvae were similarly susceptible to CA. To test whether CA could reduce *A. suum* infection in pigs *in vivo*, CA was administered daily in the diet or as a targeted, encapsulated dose. However, infection was not significantly reduced. It is proposed that the rapid absorption or metabolism of CA *in vivo* may prevent it from being present in sufficient concentrations *in situ* to exert efficacy. Therefore, further work should focus on whether formulation of CA can enhance its activity against internal parasites.

Parasitic worms of the gastrointestinal tract (helminths) are pathogens of global importance. Helminths are a major production constraint in livestock all over the world; infections result in lower growth rates and reduced milk and meat production from ruminants and pigs^1^,^2^,^3^, as well as exacerbating secondary bacterial infections^4^, leading to reduced food security and economic development. Moreover, more than a billion people, mainly in developing countries, are estimated to be infected with soil-transmitted helminths, causing substantial morbidity^5^.

With no vaccines currently available, control of helminths is based almost exclusively on mass treatment with synthetic anthelmintic drugs. This over-reliance on a limited number of chemical compounds is ultimately not sustainable^6^. Resistance to anthelmintics is widespread in livestock production systems, particularly in small ruminants where it has reached critical levels^7^, but has also been detected amongst cattle^8^ and pig^9^ parasites, and there are on-going concerns about the sustainability of mass drug administration in humans^10^. In addition, there is increasing consumer demand for animal products that are produced organically or with a minimum of synthetic chemical inputs^11^. Therefore, there is an urgent need to investigate novel approaches to helminth control.

Many plants are a rich source of bioactive compounds that can have antimicrobial and anti-parasitic effects, including essential oils and secondary metabolites. The occurrence of anti-parasitic compounds in plants allows the possibility of targeted compound identification for drug discovery, as well as the use of plants as nutraceuticals. In this way, either whole plant material or extracts are included in the diet, representing an alternative or complementary approach to parasite control, particularly in livestock^12^. This approach has many advantages, such as avoiding drug residues in agricultural products, a lower risk of parasites developing resistance, and easy integration into resource-limited communities in the developing world if and where plants are locally available^13^.

The bark of cinnamon (*Cinnamomum verum*) has been used for millennia as a traditional remedy in herbal medicine^14^. It contains high amounts of the essential oil *trans*-cinnamaldehyde (CA), as well as being a good source of proanthocyanidins (PAC), a group of plant polyphenols consisting of flavan-3-ol oligomers and polymers^15^. PAC structures vary widely depending on the degree of polymerization and nature of their flavan-3-ol subunits. The four most common flavan-3-ols are catechin and its *cis* isomer epicatechin (which give rise to procyanidin-type PAC), or gallocatechin and its *cis* isomer epigallocatechin (which give rise to prodelphinidin-type PAC). The flavan-3-ol units are linked mainly through the C_4_ → C_8_ inter-flavanol bond (B-type PAC) but flavan-3-ols can also be doubly linked by an additional ether bond between C_2_→O_7_ (A-type PAC)^16^ (Fig. 1). Cinnamon bark is relatively unusual in that it has been reported to contain PAC with a high number of A-type bonds^17^. A number of studies have investigated the anti-parasitic properties of PAC from a range of different plant sources, with reports of efficacy against both protozoan^18^,^19^ and helminth^20^ parasites, however it has not been established whether increased proportions of A- linkages in the PAC molecules can increase the potency of the anthelmintic activity. Moreover, CA has been shown to have anti-parasitic activity against *Eimeria* infections in poultry^21^, but there have been no reports on activity against helminths. Therefore, the aim of this work was to investigate the anthelmintic properties of cinnamon bark and characterise its active compounds, in order to determine if this may represent a useful natural resource for control of gastrointestinal nematodes.

## Results

### Cinnamon bark extract has potent activity against *Ascaris suum in vitro*

To test whether cinnamon bark contained compounds with anthelmintic activity, we prepared an extract using a procedure that we have previously shown to be effective in extracting polyphenolic compounds from a wide variety of plant sources^22^. The resulting extract was then tested for *in vitro* activity against larvae of *Ascaris suum*, an important nematode parasite of swine that also serves as a model for the closely related human parasite *A. lumbricoides*^23^.

**Figure 2:**
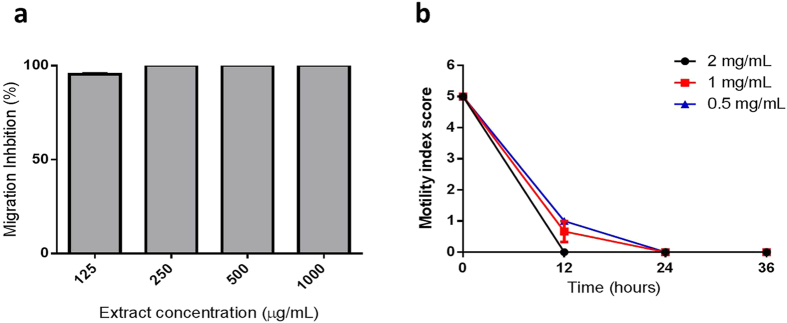
Anthelmintic effects of cinnamon bark extract against *Ascaris suum.* (**a**) Inhibition of *A. suum* third-stage larvae migration after incubation in cinnamon bark extract. Inhibition is expressed relative to larvae incubated only in culture media. Results are the means of three independent experiments, each performed in triplicate. (**b**) Inhibition of *A. suum* fourth-stage larvae motility after incubation in the cinnamon bark extract. Results are from a single experiment performed in triplicate.

We first used a migration inhibition assay to test for activity against third-stage larvae (L3), the stage of the parasite that emerges from embryonated eggs to infect the host^22^. Strikingly, overnight incubation of L3 in the cinnamon extract at concentrations between 125 and 1000 μg/mL resulted in 100% inhibition of larval migration (Fig. 2A). In contrast to this, we previously observed that extracts prepared from a number of other bioactive plant sources induced a less potent, dose-dependent inhibition of migration within the tested range of extract concentrations^22^. Subsequent observation of the larvae during incubation in the cinnamon extract revealed that at concentrations ≥250 μg/mL, all larvae died within two-three hours of incubation. We confirmed the potency of the cinnamon extract in a second experiment using fourth-stage larvae (L4) of *A. suum* recovered from the intestine of pigs after experimental infection, and again observed mortality of larvae within hours of *in vitro* incubation (Fig. 2B).

### Phytochemical analysis of cinnamon bark extract

**Figure 3:**
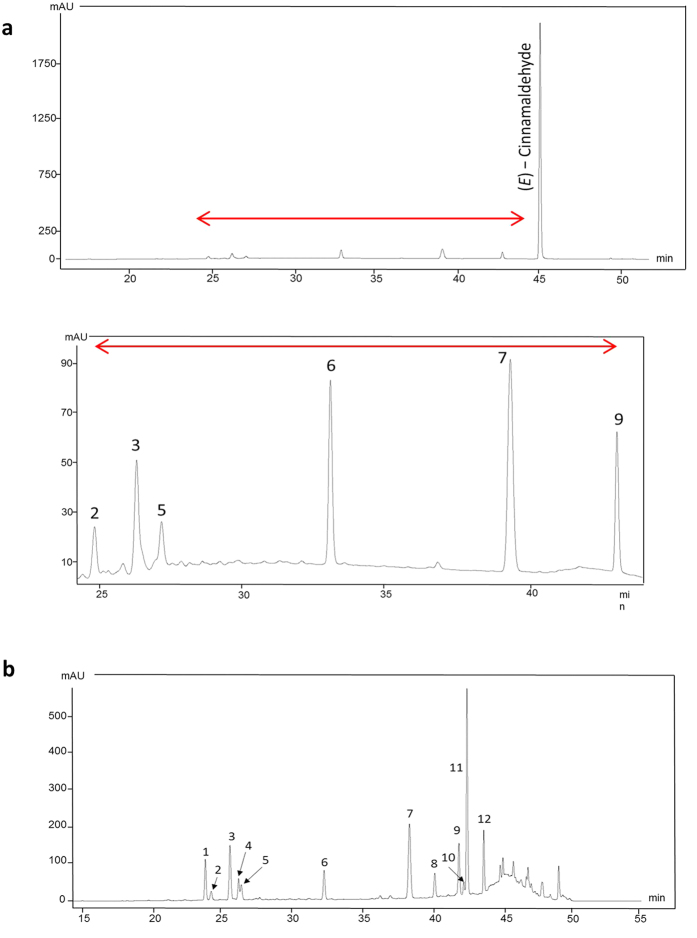
HPLC chromatograms of cinnamon bark extract. (**a**) HPLC chromatogram of cinnamon bark extract: Top panel shows detection of *trans*-cinnamaldehyde. Bottom panel shows larger version of the area indicated by the red arrow in top panel, showing **2**, A-type procyanidin (PC) dimer; **3**, A-type PC trimer; **5**, A-type PC trimer; **6**, internal standard; **7**, *cis*—Cinnamic acid; **9**, *trans*—Cinnamic acid. (**b**) HPLC chromatogram of thiolysed cinnamon bark extract (BM = benzylmercaptan adduct): **1**, Catechin; **2**, A-type PC dimer; **3**, A-type PC trimer; **4**, Epicatechin; **5**, A-type PC trimer; **6**, Internal standard; **7**, *cis*—Cinnamic acid; **8**, A-type PC—BM trimer; **9**, *trans*—Cinnamic acid; **10**, *cis* Catechin—BM; **11**, Epicatechin—BM; **12**, A-type PC—BM dimer.

To identify the putative anthelmintic compounds, we analysed the extract by LC-MS. The major compound was CA, which accounted for 7.8 g/100 g of the extract (Fig. 3A). In addition, cinnamic acid and several PAC dimers and trimers with A-type linkages were detected by LC-MS (Fig. 3A). To gain further insight into the PAC composition, the extract was subjected to thiolysis with benzyl mercaptan, which breaks PAC polymers with B-type bonds and allows quantification of their constituent flavan-3-ol units^24^,^25^. This revealed that these cinnamon PAC were procyanidins that consisted of catechin and epicatechin as terminal and extension units (Fig. 3B; Table 1). The calculated proportions of flavan-3-ol sub-units are shown in Tables S1-2. From this information, both the total amount of PAC and the mean degree of polymerization (mDP - i.e. the average length of the polymers) can be calculated. This revealed that the extract contained 24.2 g PAC/100 g extract, and the mDP was 5.2. Furthermore, analysis of the inter-flavanol links demonstrated that 21% were A-type and 79% were B-type linkages (Table 1).

To determine if the PAC were mainly responsible for the potent activity of the cinnamon extract, two PAC-enriched fractions were isolated from the extract using Sephadex LH-20 chromatography, which separated the extract on the basis of size to yield two PAC-enriched fractions (F1 and F2). The first fraction (F1) contained 52.4 g PAC/100 g of fraction, and these were lower molecular weight PAC (as determined by thiolysis; mDP of 3.7), whilst F2 contained 55.0 g PAC/100 g of fraction consisting of higher molecular weight PAC (mDP of 7; Table 1). The F1 fraction contained only trace amounts of CA, whilst CA was not detectable in the F2 fraction (data not shown).

### Activity of cinnamon bark-derived proanthocyanidins against *Ascaris suum*

**Figure 4:**
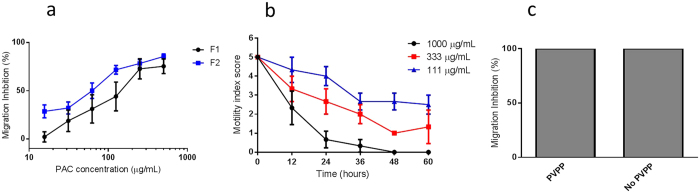
Anthelmintic effects of isolated proanthocyanidin (PAC) fractions. (**a**) Inhibition of *Ascaris suum* third-stage larvae (L3) migration after incubation in PAC fractions F1 and F2. Inhibition is expressed relative to larvae incubated only in culture media. Results are the means of two independent experiments, each performed in triplicate. Error bars indicate the inter-replicate SEM. (**b**) Inhibition of *A. suum* fourth-stage larvae (L4) motility after incubation in PAC fraction F2. Results are from a single experiment performed in triplicate. Error bars indicate the inter-replicate SEM. (**c**) Incubation in PVPP does not remove anthelmintic effects of cinnamon bark extract. Results are the means of two independent experiments, each performed in triplicate.

We next tested the F1 and F2 PAC-enriched fractions in migration inhibition assays with *A. suum* L3. In contrast to the experiments with the extract, here larvae remained alive after overnight incubation although their migratory ability was impaired (Fig. 4A), consistent with our previous study on *A. suum* L3 using a range of PAC fractions with exclusively B-type linkages^22^. Similarly, exposure of *A. suum* L4 to the PAC F2 fraction resulted in a dose-dependent decrease in motility (Fig. 4B), but the activity was far less potent than that observed previously for the whole extract (Fig. 2B). Therefore, we hypothesised that the PAC in the extract played a lesser role in the potent anthelmintic properties. To confirm this, we depleted the whole extract of PAC by overnight incubation in polyvinylpolypyrrolidone (PVPP)^26^ and repeated the L3 migration inhibition assay. Depletion of PAC did not affect the potency of the extract, whereas we previously observed that depletion of PAC from a range of other plant extracts reduced or abolished the observed anthelmintic effects^22^. Therefore, we concluded that the mixed A- and B-type PAC in the extract have comparable anthelmintic activity to the B-type PAC obtained from other plant sources^22^, and that in the case of cinnamon bark, they do not seem responsible for the potent activity of the extract.

### Anthelmintic activity of *trans*-cinnamaldehyde *in vitro*

**Figure 5:**
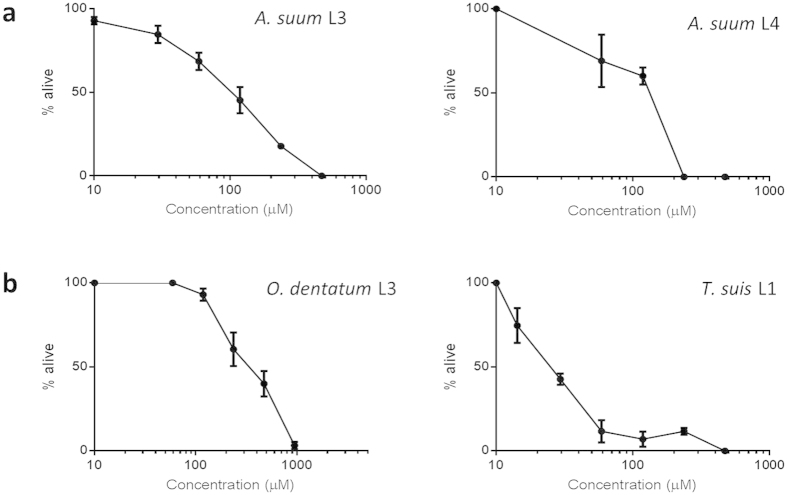
Anthelmintic effects of pure *trans-*cinnamaldehyde (CA). (**a**) Anthelmintic effects of CA against *Ascaris suum* third stage (L3) and fourth stage (L4) larvae. Mortality of *A. suum* was assessed after 12 hours incubation for L3 and 6 hours for L4. L3 results are the mean of three independent experiments, each performed in triplicate, and the L4 results are from a single experiment performed in triplicate. Error bars indicate the inter-replicate SEM. (**b**) Anthelmintic effects of CA against *Oesophagostomum dentatum* L3 and *Trichuris suis* first-stage larvae (L1). Mortality of *O. dentatum* was measured by agar-based migration inhibition assay after overnight incubation in CA, and *T. suis* mortality by observation of motility after 2 hours incubation. *O. dentatum* results are the mean of two independent experiments, each performed in triplicate, and *T. suis* results from a single experiment performed in triplicate. Error bars indicate the inter-replicate SEM.

As CA was present in relatively high concentrations in the extract, but was removed almost entirely from the PAC-fractions, we hypothesised that CA was the compound mainly responsible for the anthelmintic activity. To test this, we used pure CA (>99%) in an efficacy assay against *A. suum.* We found that pure CA had similar activity to the whole cinnamon extract against both *A. suum* L3 and L4 (Fig. 5A). Concentrations of around 200 μM (equivalent to 25.6 μg CA/mL) resulted in larval death within three hours, which corresponds very well to the amount of CA that would be present in the extract concentrations used (Fig. 2), given that 7.8% of the extract was determined to be CA. CA has been shown to be a potent antimicrobial with activity against, amongst others, *Salmonella* sp. and *Campylobacter*^27^,^28^, as well as having activity against parasitic plant nematodes^29^. However, this is the first demonstration of anthelmintic activity of CA against a gastrointestinal nematode parasite. To confirm that the activity was not only restricted to *A. suum*, we tested the activity of pure CA against *Oesophagostomum dentatum* and *Trichuris suis*, two other porcine nematodes which fall in different clades to *A. suum* and are related to human hookworm and whipworm species, respectively^30^. CA also had potent activity against larvae from these other species (Fig. 5B), demonstrating that CA has *in vitro* activity against a range of gastrointestinal parasites.

### Ultrastructural damage in *Ascaris suum* exposed to *trans-*cinnamaldehyde

**Figure 6:**
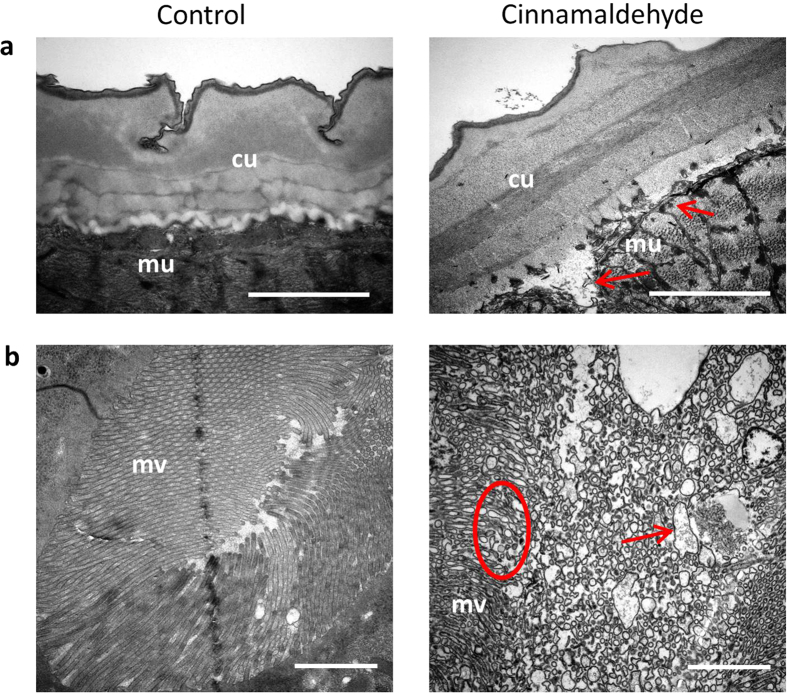
Ultrastructural changes in *Ascaris suum* exposed to *trans-*cinnamaldehyde. Transmission electron micrographs of *A. suum* fourth-stage larvae exposed to either culture media (Control) or 236 μM *trans-*cinnamaldehyde (CA) for 12 hours. For all panels scale bar indicates 2 μm. (**a**) Cuticle (cu) and underlying Muscular (mu) tissue—note the lesions in the muscle tissue underlying the cuticle and hypodermis in parasites exposed to CA (red arrows). (**b**) Digestive tissues showing the microvilli (mv) overlying the intestinal lumen—note the destruction of the villi (red circle) and the presence of large vacuoles (red arrow) in parasites exposed to CA.

To gain insight into the possible anthelmintic mechanisms of CA, *A. suum* L4 that had been exposed to a high concentration of CA (236 μM) were examined by transmission electron microscopy. Examination of the cuticle and hypodermis revealed no major changes in the CA-exposed larvae, however some localised tissue damage and lesions were observed in the muscular layer underlying the hypodermis (Fig. 6A). The most striking damage to the CA-exposed larvae occurred in the digestive tissues (Fig. 6B). Whilst control larvae had a regular, intact gut with undamaged microvilli, the same tissues were completely destroyed in larvae exposed to CA, with the microvilli having lost all integrity, and with massive lesions and vacuoles also present. Thus, it appears that damage to the internal digestive tissues may be at least partly responsible for the anthelmintic activity of CA.

### Testing of *trans*-cinnamaldehyde against *Ascaris suum in vivo*

**Figure 7:**
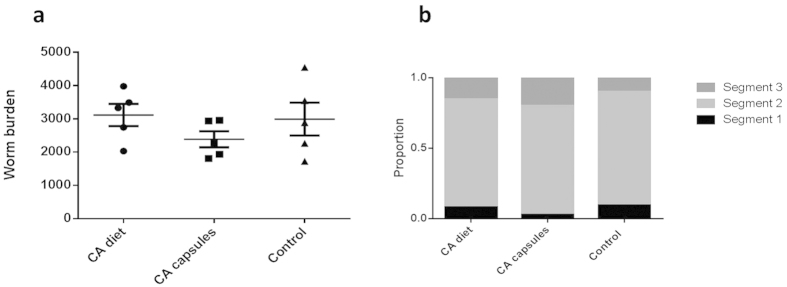
Total *Ascaris suum* larval burdens and distribution of larvae in the intestine of pigs administered *trans*-cinnamaldehyde. (**a**) Numbers of fourth-stage larvae (L4) in the small intestine (SI) at day 14 post-infection (p.i.) in pigs fed either *trans-*cinnamaldehyde (CA) in the diet daily (‘CA diet’), dosed with encapsulated CA at days 11 and 13 p.i. (‘CA capsules’), or not administered CA (‘Control’). Indicated is the mean and SEM. (**b**) Proportions of L4 recovered from the three groups in Segment 1 (proximal half of the SI), Segment 2 (third quarter of the SI) and Segment 3 (distal quarter of the SI). See materials and methods for further information.

Given that CA appeared to be the most active compound in the cinnamon extract, we proceeded to test whether pure CA could reduce infection with *A. suum* in pigs *in vivo.* Two different approaches were taken to administer CA; as a daily dietary supplement or as a targeted therapeutic dose in encapsulated form, as this latter approach may be 1) more suitable to deliver a concentrated dose of the active compound to the site of infection (the small intestine for *A. suum*), and 2) may be more applicable to human populations where a therapeutic dose needs to be applied rather than a preventative dietary approach. Thus, three groups of pigs were used. The first group remained untreated as a control. A second group of pigs was fed a daily supplement of 1000 mg CA, as this dose has been shown to be safe and acceptable to pigs, as well as resulting in decreased *Escherichia coli* excretion^31^. Five days after the supplemental feeding began, all three groups of pigs were infected orally with 5000 embryonated *A. suum* eggs. The third group was then orally dosed with 1000 mg of CA, placed in acid-resistant capsules, on two occasions, 11 and 13 days post-infection. At 14 days post-infection, all pigs were killed and larval burdens were enumerated. Pigs that received the CA capsules had a mean larval burden of 2386, compared to 3114 and 2991 in those that received the dietary CA or no treatment, respectively. However, there were no significant differences (Fig. 7A; *P* = 0.28 by one-way ANOVA). We also assessed the location of larvae within the small intestine, to determine if CA treatment resulted in a more posterior location of larvae. The majority of larvae (~80%) were found in the third quarter of the SI in all pigs. Pigs dosed with CA capsules had more larvae located in the fourth (most posterior) quarter (20%) compared to the control (10%) or those fed CA in the diet (15%), however these differences were also not significant (Fig. 7B; *P* = 0.3 by one-way ANOVA). Therefore, despite its potent *in vitro* activity, CA failed to have an *in vivo* effect in this model of *A. suum* infection.

## Discussion

We found that the *C. verum* extract contained PAC with a mixture of A- and B-type linkages, consistent with a previous report on the composition of PAC in *C. zeylanicum* bark^17^, however these authors also noted the presence of prodelphinidins, which were not observed in our current study, where PAC were comprised exclusively of procyanidins. A number of groups have investigated the anthelmintic properties of PAC, and indeed we recently reported that PAC derived from a wide variety of plant sources had strong anthelmintic activity against *A. suum in vitro*^22^. However, these experiments were performed with PAC that were comprised largely of polymers with B-type linkages. As A-type linkages markedly affect the 3-dimensional structure and rigidity of PAC molecules, we were interested to ascertain whether they may enhance anthelmintic activity. Indeed, a recent report has indicated that increased proportions of A-type linkages may enhance the *in vitro* activity of PAC against *E. coli*^32^. The potent, lethal effect of the extract tested here against *A. suum* indicated the presence of other compounds with superior anthelmintic activity to the range of PAC molecules which have been tested previously against a multitude of helminths including *A. suum*, *O. dentatum*^33^, *Ostertagia ostertagi*^34^ and *Haemonchus contortus*^35^. This rapid lethality also contrasted to our previous experiments with *A. suum* larvae where incubation in other tested plant extracts, or synthetic anthelmintic drugs such as ivermectin, resulted in impaired motility and migratory ability but generally larvae remained alive (data not shown). However, we established that this potency did not derive from the PAC molecules in this cinnamon extract. Thus, we concluded that the proportion of A- and B-type linkages in PAC molecules does not markedly influence anthelmintic activity, at least in our *in vitro* experiments with *A. suum.* Instead, our data strongly pointed towards CA as responsible for the potent anthelmintic properties, with lethal *in vitro* activity towards a range of different gastrointestinal nematodes, and that the mechanism appears to involve the physical destruction of the intestinal tissue of the parasite. This presumably occurs after ingestion of the compound from the surrounding media, as the integrity of the cuticle did not seem to be noticeably compromised. The exact mechanism of the anthelmintic effect can only be speculated upon, but CA being an α, β unsaturated aldehyde can react with various nitrogen groups and interference with key enzymes and subsequent cell lysis has been proposed as an antibacterial mechanism of CA^36^,^37^. Similar mechanisms may operate against nematode parasites.

Despite these promising *in vitro* data, we did not observe any *in vivo* activity when CA was administered to pigs, either as a daily dietary supplement or in encapsulated form by gavage. CA has recently been investigated as a daily feed supplement for pigs and poultry, and some anti-bacterial efficacy has been reported with this strategy^31^,^38^. Several reasons may exist why we did not detect any anthelmintic activity in our *in vivo* model. First, a higher dose may be required to achieve *in vivo* efficacy. Whilst dose-response studies were outside of the scope of this study, the dosage used in the dietary supplement (1000 mg per pig per day, equivalent to approximately 35 mg/kg bodyweight) was chosen based on previous reports showing that this dose was well tolerated by pigs and reduced *E. coli* excretion^31^,^39^. Moreover, assuming a small intestinal volume of approximately 2L, we assumed that a dose of 1000 mg would result in approximately the same concentrations in the intestine that resulted in efficacy in our *in vitro* studies, even assuming for a large degree of compound disappearance (up to 90%) during digestion. Toxicity studies for CA do not exist for pigs, but rodent studies indicated that the LD_50_ for CA given daily by gavage is in the region of 2500 mg/kg bodyweight/day—some 100-fold higher than we used here^40^. Therefore, we considered our current dosage to be towards the maximum end of what can be safely administered, allowing a wide safety-margin which is necessary for future usage. Second, a previous study has shown a similar discrepancy between the *in vitro* effect and *in vivo* responses in using CA to treat *Salmonella* in pigs^41^. These authors showed that this may be due to the non-specific binding of CA to elements of the pig diet formula reducing *in vivo* availability. Certainly, CA is a reactive molecule, and is not only specific for pathogens but will combine with many other endogenous molecules such as proteins in mucus or digesta, particularly those with thiol-containing amino acids^42^. Lastly, and what we consider most likely, the rapid absorption or metabolism of CA may prevent it from reaching the intestinal site of the nematodes in sufficient concentrations to exert *in vivo* efficacy. Michiels *et al.* have shown that the vast majority of CA is absorbed from the stomach and the proximal duodenum in pigs^39^. Whilst *A. suum* larvae pass through the stomach to complete their migratory phase after passing through the lungs and liver, it is unlikely that there will be sufficient contact between the CA and the parasite before the majority of the compound disappears through the stomach. To try and circumvent this problem, we included an additional group in the *in vivo* study that was dosed with CA in acid-resistant capsules that are designed to protect the dose through the stomach and release it in the small intestine. However, given that the *A. suum* infection was already established within the intestine and rapidly moving to a posterior location (as noted in our post-mortem data where only a tiny proportion of larvae were found in the anterior half of the small intestine at day 14 post-infection), it may be that even this approach is not suitable for delivering a sufficiently high dose to the site of the infection. However, it may be interesting to ascertain whether CA could have *in vivo* efficacy on *A. suum* adults, which reside in the anterior half of the small intestine^43^.

Whilst the rapid absorption of CA from the stomach reduces its potential as an anthelmintic agent against intestinal-dwelling parasites, formulation of the compound to increase its stability may present an option to overcome this issue. Notably, Si *et al.* have shown that emulsification of essential oils (including CA) in a variety of hydrocolloids can restore *in vitro* antimicrobial activity which is lost in the presence of pig dietary compounds^41^. Therefore, experiments to determine whether emulsification/stabilisation agents can enhance the *in vivo* anthelmintic activity of CA are warranted, as are studies against parasites residing in the anterior regions of the small intestine. Moreover, optimisation of microencapsulation procedures to protect the compound and facilitate its slow release in the intestine may also be a suitable option.

In conclusion, we have shown for the first time that cinnamon bark has anthelmintic potential *in vitro*, and this derives both from its proanthocyanidin tannins and most notably from *trans*-cinnamaldehyde. However, for the potential of *trans-*cinnamaldehyde to be realised as an anthelmintic against intestinal helminths *in vivo*, appropriate formulations to stabilise and protect the compound will likely be necessary.

## Materials and Methods

### Ethics statement

All animal experimentation was approved by the Experimental Animal Unit, University of Copenhagen, and carried out according to the guidelines of the Danish Animal Experimentation Inspectorate (Licence number 2010/561-1914).

### Experimental Design

**Figure 8:**
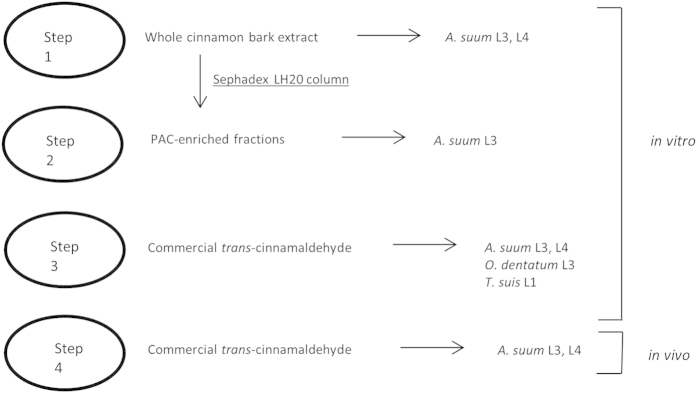
Schematic experimental design. Stepwise outline of the anthelmintic testing of cinnamon bark extract, derived proanthocyanidin fractions and pure *trans*-cinnamaldehyde.

A schematic outline of the experimental design for the anthelmintic testing is presented in Fig. 8. Cinnamon bark extract was first extracted and tested against *A. suum in vitro* using migration and motility inhibition assays. Two PAC fractions were derived from the extract and also tested against *A. suum in vitro.* Pure CA was then purchased commercially and tested against *A. suum, O. dentatum* and *T. suis* larvae *in vitro* and *A. suum* larvae *in vivo.*

## Materials

Cinnamon bark (*Cinnamomum verum*) was obtained from Dary Natury (Grodzisk, Poland). Hydrochloric acid (36%), acetone [analytical reagent (AR) grade], dichloromethane (HPLC grade), and methanol (HPLC grade) were purchased from ThermoFisher Scientific Ltd (Loughborough, UK); (±)-taxifolin (98%) from Apin Chemicals (Abingdon, UK); benzyl mercaptan (98%) from Sigma-Aldrich (Poole, UK) and procyanidin A2 (≥99% HPLC) from Extrasynthèse (Genay Cedex, France). Deionised water (dH_2_O) was purified in an Option 3 water purifier (ELGA Process Water, Marlow, UK) and ultrapure water was obtained from a Milli-Q System (Millipore, Watford, UK). For analytical experiments, cinnamaldehyde (95%) was obtained from Sigma-Aldrich (Poole, UK) and for anthelmintic studies *trans*-cinnamaldehyde (>99%) and PVPP were obtained from Sigma-Aldrich (Schellendorf, Germany).

### Extraction procedure and purification of proanthocyanidins

Finely ground cinnamon bark (10 g) was extracted with acetone/water (140 mL, 7:3, v/v) for 1 h to yield a crude extract. Acetone was removed under vacuum on a rotary evaporator at 35 °C; the remaining aqueous solution was centrifuged for 3 min at 3000 rpm and freeze-dried to yield 2.25 g. The crude cinnamon extract (940 mg) was dissolved in distilled water (500 mL) and applied to a Sephadex LH-20 column (20 g, GE Healthcare, Little Chalfont, UK), which was conditioned with water. Deionised water (1 L) was added to remove sugars. Then, the first fraction (F1-fraction) of purified tannins was eluted with acetone/water (1 L, 3:7, v/v) and the second fraction (F2-fraction) was eluted with acetone/water (1 L, 1:1, v/v). The acetone was removed on a rotary evaporator at 35 °C and the remaining aqueous solutions were frozen overnight and freeze-dried.

### LC-MS analysis

Thiolysis reactions were performed as previously described^44^, except that 8 mg of extract or fraction was used for the reaction. Flavan-3-ols and their benzyl mercaptan (BM)-adducts were identified by LC-MS analysis on an Agilent 1100 Series HPLC system and an API-ES instrument Hewlett Packard 1100 MSD detector (Agilent Technologies, Waldbronn, Germany). Samples (20 μL) were injected into the HPLC connected to an ACE C_18_ column (3 μm; 250 × 4.6 mm; Hichrom Ltd; Theale; UK), which was fitted with a corresponding ACE guard column, at room temperature. The HPLC system consisted of a G1379A degasser, G1312A binary pump, a G1313A ALS autoinjector and a G1314A VWD UV detector. Data were acquired with ChemStation software (version A 10.01 Rev. B.01.03). The flow rate was 0.75 mL min^−1^ using 1% acetic acid in water (solvent A) and HPLC-grade acetonitrile (solvent B). The following gradient program was employed: 0–35 min, 36% B; 35–40 min, 36–50% B; 40–45 min, 50–100% B; 45–55 min, 100–0% B; 55–60 min, 0% B. Eluting compounds were recorded at 280 nm. Mass spectra were recorded in the negative ionization scan mode between *m/z* 100 and 1000 using the following conditions: capillary voltage, −3000 V; nebulizer gas pressure, 35 psi; drying gas, 12 mL min^−1^; and dry heater temperature, 350 °C. Flavan-3-ol terminal and extension units were identified by their retention times and their molecular masses and their peak areas at 280 nm were integrated and quantified using response factors relative to taxifolin^25^. A response factor of 0.55 for A-type procyanidin dimer was calculated using authentic procyanidin A2, and in the absence of commercially available trimers the same response factor was used for A-type procyanidin trimers and their benzylmercaptan adducts (Ropiak *et al.,* submitted). This provided information on the PAC composition in terms of % terminal and % extension flavan-3-ol units. It also allowed calculation of % procyanidins and % *cis*- and *trans-*flavan-3-ols according to Gea *et al.*^25^, excluding the *cis*/*trans* ratio for A-type PAC. The percentage of A-type linkages (% A-type) and mean degree of polymerization (mDP) were calculated according to Ropiak *et al.* (submitted). For quantification of CA, pure CA was dissolved in methanol/water (1:1) at a range of concentrations between 0.03 and 0.13 g/L in order to generate a standard curve from the corresponding peak areas. The whole extract was analysed under identical conditions and the concentration of CA in the extract determined by extrapolation from the standard curve.

### Parasites

*A. suum* eggs were obtained from gravid adult worms collected from a local slaughterhouse (Danish Crown, Ringsted, Denmark) and embryonated and stored as described^45^. To obtain the L3, eggs were hatched by mechanical stirring with 2 mm glass beads for 30 minutes at 37 °C, and then viable larvae were purified by incorporating the eggs/hatched larvae into 0.5% agar and incubating overnight at 37 °C in RPMI 1640 media containing L-glutamine (2 mM), penicillin (100 U/mL), streptomycin (100 μg/mL) and amphotericin B (0.5 μg/mL). Larvae that migrated into the media were collected and used in the subsequent anthelmintic assays. L4 were collected from the small intestine of pigs 12–14 days after experimental infection, purified from the gut content and washed as described previously^22^. *O. dentatum* eggs were obtained by collecting faeces from mono-infected donor pigs, and L3 were produced by copro-culture and stored in water at 10 °C. *T. suis* L1 were produced by mechanical hatching of embryonated eggs, followed by collection of viable larvae after migration through a 20 μm sieve, as previously described^46^.

### Anthelmintic assays

Migration and motility assays for *A. suum* were conducted as previously described^22^. Briefly, for the migration inhibition assay, 100 *A. suum* L3 were added in triplicate to 48-well plates and incubated (37 °C, 5% CO_2_ in air) overnight in either cinnamon extract, PAC fractions or CA. Agar was then added to a final concentration of 0.8% and the number of larvae able to migrate was assessed by light microscopy. In addition, the survival of L3 exposed to either cinnamon extract or CA was assessed at hourly intervals after the start of the incubation. Larvae were considered alive if they had a characteristic coiled appearance and were motile, and were considered dead if straight and immobile even after extended observation^47^. For PAC-depletion experiments, the extract (1 mg/mL) was incubated overnight at 4 °C with PVPP at a ratio of 1 mg extract to 50 mg PVPP. The extract was then centrifuged for ten minutes at 3000 g and the supernatant used in the assays. Controls consisted of media alone with PVPP and whole extract with no PVPP that were incubated in an identical fashion.

Motility of L4 exposed to either cinnamon extract or PAC fractions was assessed at 12-hour intervals for up to 60 hours using a motility scoring system where 5 is fully motile and 0 is no movement^22^,^48^. Larvae that scored 0 on two successive time-points were considered dead. The motility of L4 exposed to CA was assessed after 6 hours incubation. All assays included media only, as a negative control, and ivermectin (50 μg/mL) as a positive control. *O. dentatum* L3 survival was also assessed by an agar-based migration inhibition assay^33^. The number of migrating worms was used as a surrogate measure for the number of live worms, as we have observed with *O. dentatum* in this assay that close to 100% of viable worms are able to migrate. Survival of *T. suis* exposed to CA was assessed as described above for survival of *A. suum* L3, after 2 hours incubation.

### Electron Microscopy

Transmission electron microscopy was carried out as described previously^22^. Briefly, worms were washed well in PBS and fixed in 2% glutaraldehyde in 0.05 M phosphate buffer. Samples were post-fixed and dehydrated before sectioning at 70 nm. Imaging was done using a Phillips CM100 microscope. Images were obtained with an Olympus Veleta camera and processed using ITEM software.

### *In vivo* testing

Fifteen helminth-naive pigs (Danish Landrace/Yorkshire/Duroc, mean weight 26 kg) of mixed sex (females and castrated males) were obtained from a specific-pathogen free (SPF) farm with no history of helminth infection. The animals were stratified on the basis of sex and weight to three groups (n = 5) and housed in separate pens with concrete floors. Straw was provided daily. All the pigs were fed restrictively (1 kg per pig/day) with a diet consisting of 75% ground barley and 25% protein/mineral supplement (NAG, Helsinge, Denmark) and had free access to water. Group 1 served as a control. Group 2 was fed a daily supplement of 1000 mg cinnamaldehyde/kg of feed, which was mixed thoroughly into the feed immediately prior to feeding. Five days after the commencement of the supplementary feeding, all 15 pigs were inoculated with 5000 *A. suum* eggs by stomach tube. On days 11 and 13 post-infection (p.i.), a time-point when the larvae had returned to the small intestine following migration, pigs in group 3 were administered 1000 mg of cinnamaldehyde encapsulated in acid-resistant gelatin capsules (size 2 DR capsules, Capsugel, Bornem, Belgium) directly into the stomach by gavage. Pigs in the control group were similarly administered empty capsules by gavage on days 11 and 13 p.i. On day 14 p.i. all pigs were killed by captive bolt pistol and exsanguation. The entire small intestine was removed, and separated into equal halves. The distal half of the intestine was further separated into two equal segments. Thus, three segments of the small intestine were obtained (first half, third quarter and fourth quarter), and worms were isolated from a 50% subsample of each of these segments separately by the agar-gel method using two hours of incubation^49^. Worms were stored in 70% ethanol and then counted using a dissecting microscope.

### Data analysis and statistics

Where mentioned, ANOVA analyses were carried out using GraphPad Prism version 6.0.

## Additional Information

**How to cite this article**: Williams, A. R. *et al.* Anthelmintic activity of *trans*-cinnamaldehyde and A- and B-type proanthocyanidins derived from cinnamon (*Cinnamomum verum*). *Sci. Rep.* **5**, 14791; doi: 10.1038/srep14791 (2015).

## Supplementary Material

Supplementary Information

## Figures and Tables

**Figure 1:**
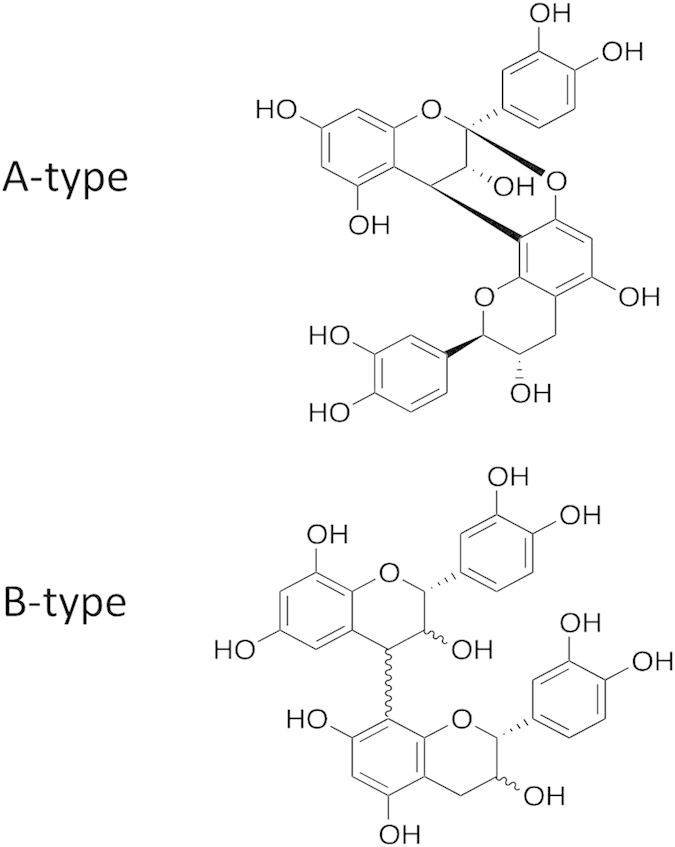
Examples of an A-type and a B-type procyanidin dimer.

**Table 1:** Proanthocyanidins (PAC) in cinnamon bark extract and purified fractions.

Cinnamon	PAC	mDP	A-type %	PC	cis*	trans*
Extract	24.2 (0.3)	5.2 (0.0)	21.3 (0.2)	100.0 (0.0)	66.6 (0.2)	33.4 (0.2)
F1-fraction	52.4 (1.0)	3.7 (0.0)	37.4 (0.1)	100.0 (0.0)	72.9 (0.2)	27.1 (0.2)
F2-fraction	55.0 (0.9)	7.0 (0.1)	18.1 (0.3)	100.0 (0.0)	85.9 (0.1)	14.1 (0.1)

PAC content (g of flavan-3-ol/100 g DW), mean degree of polymerization (mDP), PAC composition [% A-type linkages—remainder is B-type linkages; % procyanidins (PC); % *cis-* and *trans-*flavan-3-ols].

^*^Does not include flavan-3-ols in A-type PAC.
