# Supplementary material for: Anthelmintic activity of trans-cinnamaldehyde and A- and B-type proanthocyanidins derived from cinnamon (Cinnamomum verum)
Source: Sci Rep. 2015 Sep 30;5:14791. doi: 10.1038/srep14791 (PMC4588565; doi:10.1038/srep14791)
Supplement: Supplementary Information [file srep14791-s1.pdf]

Supplementary Material:

Anthelmintic activity of *trans*-cinnamaldehyde and A- and B-type proanthocyanidins derived from cinnamon (*Cinnamomum verum*)

Andrew R. Williams, Aina Ramsay, Tina V.A. Hansen, Honorata M. Ropiak, Helena Mejer, Peter Nejsum, Irene Mueller-Harvey, Stig M. Thamsborg

Supplementary Table 1: Composition of flavan-3-ol terminal units of extract and purified fractions of proanthocyanidins (PAC) of cinnamon in relative percentage of total PAC (%) and mg of flavan-3-ols/g DW.

**Cinnamon**	**Terminal units**									
	**HPLC peak 1**		**HPLC peak 2**		**HPLC peak 3**		**HPLC peak 4**		**Peak 5**	
	%	mg/g	%	mg/g	%	mg/g	%	mg/g	%	mg/g
Extract	9.5 (0.1)	23.0 (0.2)	1.4 (0.1)	3.4 (0.1)	8.0 (0.1)	19.4 (0.1)	4.7 (0.1)	11.4 (0.1)	2.2 (0.1)	5.3 (0.1)
F1-fraction	13.4 (0.1)	70.2 (0.3)	2.9 (0.1)	15.2 (0.1)	14.3 (0.1)	74.9 (0.6)	7.0 (0.1)	36.7 (0.1)	4.5 (0.1)	23.6 (0.7)
F2-fraction	6.8 (0.1)	37.4 (0.1)	1.8 (0.1)	9.9 (0.1)	5.7 (0.2)	31.4 (0.1)	3.1 (0.1)	17.1 (0.1)	1.2 (0.1)	6.6 (0.1)

Supplementary Table 2: Composition of flavan-3-ol extension units of extract and purified fractions of proanthocyanidins (PAC) of cinnamon in relative percentage of total PAC (%) and mg of flavan-3-ols/g DW.

**Cinnamon**	**Extension units**							
	**HPLC peak 8**		**HPLC peak 10**		**HPLC peak 11**		**HPLC peak 12**	
	%	mg/g	%	mg/g	%	mg/g	%	mg/g
Extract	3.3 (0.1)	8.0 (0.1)	16.9 (0.3)	40.9 (0.4)	47.9 (0.8)	115.9 (0.1)	6.2 (0.1)	15.0 (0.1)
F1-fraction	1.9 (0.1)	10.0 (0.1)	4.0 (0.1)	21.0 (0.6)	39.8 (0.4)	208.6 (0.5)	12.4 (0.1)	65.0 (0.1)
F2-fraction	4.6 (0.1)	25.3 (0.1)	4.6 (0.2)	25.3 (0.2)	66.6 (1.1)	366.3 (0.1)	5.5 (0.1)	30.3 (0.1)
